# Homeless drug users' awareness and risk perception of peer "Take Home Naloxone" use – a qualitative study

**DOI:** 10.1186/1747-597X-1-28

**Published:** 2006-10-02

**Authors:** Nat Wright, Nicola Oldham, Katharine Francis, Lesley Jones

**Affiliations:** 1 Healthcare Department, HMP Leeds, Leeds, UK; 2 NFA Health Centre for homeless people, Leeds, UK; 3 Department of Medicine, Kings College London, UK; 4 Centre for Research in Primary Care, Leeds, UK; 5 HMP Leeds Health Care Department, 2 Gloucester Terrace, Armley, Leeds, LS2 9PL, UK

## Abstract

**Background:**

Peer use of take home naloxone has the potential to reduce drug related deaths. There appears to be a paucity of research amongst homeless drug users on the topic. This study explores the acceptability and potential risk of peer use of naloxone amongst homeless drug users. From the findings the most feasible model for future treatment provision is suggested.

**Methods:**

In depth face-to-face interviews conducted in one primary care centre and two voluntary organisation centres providing services to homeless drug users in a large UK cosmopolitan city. Interviews recorded, transcribed and analysed thematically by framework techniques.

**Results:**

Homeless people recognise signs of a heroin overdose and many are prepared to take responsibility to give naloxone, providing prior training and support is provided. Previous reports of the theoretical potential for abuse and malicious use may have been overplayed.

**Conclusion:**

There is insufficient evidence to recommend providing "over the counter" take home naloxone" to UK homeless injecting drug users. However a programme of peer use of take home naloxone amongst homeless drug users could be feasible providing prior training is provided. Peer education within a health promotion framework will optimise success as current professionally led health promotion initiatives are failing to have a positive impact amongst homeless drug users.

## Background

Heroin overdose is a major cause of death amongst problematic drug using populations [1]. Worldwide, such drug related deaths (DRDs) are common and increasing [2,3] Heroin related overdose is more common amongst drug users who are not actively engaged in drug treatment services [2]. It is strongly associated with marginalisation and exclusion as many drug users are reluctant to call for an ambulance when a fellow drug user overdoses [4]. Fear of police involvement has been described as the principal reason for their reluctance to involve emergency paramedic staff [4,5]. As a result, ineffectual techniques have sometimes been used by drug users attempting to resuscitate the comatose drug user. Such techniques involve injecting the user with saline, inflicting pain, walking the patient round the room and shouting at the person in an attempt to reverse coma [4]. It is in this context that, internationally, the concept of "take home naloxone" (THN) has been discussed. There are differing models of naloxone provision to drug users related to the intensity of training and follow-up. In the USA, some states have enacted legislation to permit persons other than than licensed medical providers to administer naloxone in an emergency situation providing they have undertaken a formal training programme [6,7]. Peer use of naloxone is carefully audited [8]. This model is similar to a pilot project undertaken in Berlin, Germany in 1999. In the project users attending a mobile healthcare project were offered training in emergency resuscitation after overdose, provided with naloxone, needles, syringes, an emergency handbook and information on naloxone [9]. In the same paper there was a brief account of a pilot project in Jersey where naloxone was provided to users attending health services with instructions on intramuscular use and also wider principles of resuscitation. By contrast, in Italy, naloxone has been available "over the counter" in pharmacy stores since 1995 though there appears to be a paucity of research evaluating the effectiveness of this model [7]. Arguably there is a trade-off as there is a possibility for wider coverage of at risk populations by making naloxone available over the counter. However providing naloxone from health or drug services permits more intensive training and follow-up. However it limits the intervention to those users presenting to treatment services. Such issues are of relevance to homeless drug users, many of whom are excluded from drug treatment services yet are at high risk of heroin overdose.

The concept of THN for peer use by drug users has been widely debated. In the United Kingdom there has recently been a change in Statute regarding the status of take home naloxone. Although the drug remains a prescription only medicine, a recent Statutory Order which came into force on June 30^th ^2005 permits any third party to administer the drug to another human being in the event of an emergency [10]. Such a change has provided the legal framework for the provision of THN in the UK setting. However a professional framework for the administration of THN in the primary care setting has yet to be developed [11]. Due to the recent large expansion in the number of health care staff working with drug users in the primary care setting [12] and the growth in both mainstream and "specialised" primary care practices working with homeless people^15 ^consideration of THN amongst homeless populations is timely.

In the context of this study homelessness refers to the lack of a "decent, secure, affordable home within a strong community"[13]. In the UK setting, periods of homelessness are often episodic, with housing careers characterised by frequent movement between different types of housing [14]. Drug dependence is the major expressed health need of such homeless populations [14], who represent the most marginalized sub-group of the drug using population [15,16]. This study explored the relationship between acceptability to drug users, the degree of responsibility that drug users are prepared to take in the overdose situation and a description of possible risk entailed in introducing such a project. Risk can be defined as "the probability that a hazard will give rise to harm"[17]. Different people have differing perceptions of risk according to how they both construct their reality and evaluate risks according to subjective perceptions [18]. Additionally the balance between benefit and harm of any health intervention (including THN) is neither simple nor static [19]. This study sought to explore such inter-relationships between risk perception and possible benefits or harms of THN amongst homeless drug users. From the findings of this study we seek to recommend which model of THN provision would be most appropriate for the needs of homeless drug users.

## Methods

The study was conducted across three sites in one centre. The centre was a large cosmopolitan city of 750,000 residents which has a large homeless population. Amongst this population there is a particularly high use of illicit heroin and crack cocaine. All three sites provided services to homeless drug users. One was a primary care centre and two were non-statutory organisations.

Ethical approval for the study was obtained from the National Health Service Local Research Ethics Committee. Recruitment of participants was by poster advertisement, contact through key workers or by personal approach from the researcher through the participating agencies. All respondents were given verbal and written information about the project and signed a consent form after which a food voucher was given as an incentive to complete the interview.

The study group comprised 19 men and 8 women. The ethnic background of all respondents was white Caucasian. The age range was 21–58 years old. A purposive sampling strategy was developed to recruit a mixed group of respondents. Inclusion criteria were past or current history of heroin use, past or current history of homelessness, and either personal experience or experience of a peer having taken a heroin overdose. Drug users were excluded from the study if they were intoxicated at the time of interview.

The range of accommodation at the time of interview is described in table 1. The majority of participants had used heroin for more than two years. One respondent reported they had not used heroin within the last 12 month period.

The interviews were carried out by an independent researcher (NO) who was previously unknown to the respondents. Interviews lasted up to 90 minutes. They were semi-structured and naturalistic, using a topic guide based on aspects of housing and its impact upon risk factors for heroin overdose. Such risk factors included their own personal experiences of heroin overdose, as well as those of their peers, the type of accommodation (and its impact upon heroin use), and any factors that might increase the likelihood of solitary injecting. These findings have been reported elsewhere [20]. Additionally the following topic areas were explored: ability to recognise the signs of an overdose and subsequent actions taken; awareness of naloxone and attitudes towards naloxone distribution and use. The findings are presented in this paper.

Twenty seven people were interviewed. Two interviews were discarded, one because the respondent was intoxicated at the time of interview, the other due to poor quality recording.

All the interviews took place over a 15 month period between 2002–2003. Interviews were audio recorded in a private room in a setting familiar to the respondent.

Each interview was transcribed and analysed by two independent researchers (NO & KF).

A thematic analysis approach was employed for the analysis of the transcripts which involved developing core concepts, categories and themes. Central to this method was the process of collaborative coding whereby categories, themes and codes were constantly developed and compared throughout the analytical stages by two independent researchers searching for anomalies and disconfirming data not representative of a theme [21]. Where the two independent researchers developed different interpretations of the data, conflict was resolved by discussion with two further independent researchers (NW and LJ). Using such a framework ensured consensus regarding core coding categories, themes and use of illustrative quotes that were representative of the data collected. Data collection stopped once no new data emerged from analysis of the interviews.

## Results

Direct quotes from participants are reported below to support the findings. Pseudonyms are used to protect confidentiality with the participant's age, length of heroin use and accommodation status shown in brackets after the pseudonym.

### Peer response to a heroin overdose

For peer users to appropriately perform resuscitation and administer THN in the event of a heroin overdose, users would need to be able to correctly identify the signs of an overdose as distinct from a state of heroin induced intoxication. Administering THN to those who were intoxicated would have the potential to harm the credibility of the programme as it would induce a state of acute opiate withdrawal.

#### Recognising the signs of overdose

Participants were clearly able to distinguish between a state of intoxication and a state of coma due to heroin overdose. Such physical indicators included cyanosis, unconsciousness (from which they cannot be roused by inflicting pain or physical force) and respiratory depression:

"When someone gets like, goes kinda blue and stuff and makes funny noises and you can't even bring them round, even if you are shaking them and hitting them, you can't bring them round." (Mark, 37 years, 20 year history, living with a friend)

"I'd seen like he'd gone...[participant makes the sound of gasping breath] really shallow, a lot of breaths and that lot and then a dodgy gasp of breath and that were it. It were all over he went blue." (Graham, 23 years, 10 year history, residential rehabilitation)

The notion of personal responsibility towards peers in the event of an overdose was explored. It was felt that if they currently considered themselves to have a 'duty of care' towards their peers, then such a sense of responsibility could be the foundation for a programme of peer administration of THN. Many respondents described situations where they had become concerned for a peer's state of health and tried to resuscitate the individual. Many participants appeared willing take responsibility and be proactive in taking measures to revive and resuscitate a fellow user in the event of an overdose. However actions often included both established CPR methods and ineffectual methods. Such methods included walking the overdose victim around the room, inflicting pain by slapping/hitting him/her or attempting to perform cardiopulmonary resuscitation (CPR). Users were more likely to report attempting cardiac resuscitation rather than respiratory resuscitation despite the fact that heroin is more likely to cause respiratory depression:

"Just chest compressions and I have poured water on them, and just kept talking to them. Slapped their faces a little bit, not violently, in an attempt to bring them round. You know whatever, anything they can do to just try and wake up." (Andy, 35 years, 10 year history, sleeping rough)."

#### Barriers to taking responsibility

Users' willingness to take responsibility appeared to be context dependent. They were more reluctant to take responsibility if saving someone's life involved calling an ambulance as it increased the possibility of police involvement. Fear of police attending an overdose scene would for some people be a deterrent to them calling the emergency services:

"Some people panic obviously and get the hell out of there. Some people just waited around because they had nothing to do with them, urr yeah you are right most people do panic and the fear of the police and all that carry on and get the hell out of there, so I have seen people disappear when people have gone over before." (Jack, 30 years, 8 year history, council property)

Users also appeared reluctant to take responsibility if an overdose occurred in the hostel setting. They were concerned that being associated with group drug using behaviour could lead to eviction and loss of tenancy if such behaviour became known to hostel staff.

"They actually told a member of staff and I actually got a warning for it. If anything happened like that again or if we were using the stuff in the hostel and all that I would be thrown out." (Chris, 32 years, 6 year history, sleeping rough)

Lack of knowledge regarding how to act appropriately in the overdose situation prevented users from taking responsibility. A theme emerged of abdicating responsibility and leaving the scene being preferable to staying in the event of an overdose and not being able to take appropriate actions of resuscitation:

"And I was gone me, I was scared to tell you, if he had died it wouldn't have been my fault, I didn't give it (heroin) to him but I was there, you know what I mean. I would have been faced with the guilt that I had not done what I was supposed to have done." (Claire, 25 years, 9 year history, hostel accommodation).

### Awareness and risk perception of peer THN use

The research project entailed exploring participants' knowledge regarding the mechanism of action of naloxone, their attitudes towards either giving or receiving naloxone, their beliefs regarding the potential for misuse or malicious use and their attitudes towards calling for the help of emergency services after having administered the drug.

#### User knowledge regarding THN

Having an understanding of drug users' prior knowledge of THN will help inform future training programmes. Clearly if drug users have limited knowledge regarding the drug then it would be difficult to recommend naloxone be made available "over the counter" to drug users. Respondents varied in the degree of prior knowledge that they had regarding naloxone. Many drug users had good prior working knowledge demonstrating that any future programme of "take home naloxone" would not be entirely alien to homeless drug users. Knowledge appeared to have been acquired either from the influence of the media, from personal experience of having received naloxone, or from other users' recounting their experience of receiving naloxone from ambulance staff. They were aware of the immediate onset of action of naloxone in reversing respiratory depression:

"Well it's a short acting opiate blocker isn't it? It's not like the adrenaline in the heart on Pulp Fiction. It's a quick acting blocker but short term. It's not like Naltrexone that stays in you for three days, so you're not doing mad turkey in hospital. You can still sort yourself out can't you, a couple of hours later?" (Peter, 34 years, 21 year history, living with his sister)

"The ambulance comes out they give you something called narcan or something or other, an injection or something." (Laura, 23 years, 10 year history, hostel accommodation)

The need for prior training in the pharmacological properties of heroin and its action upon the body's physiology was identified. Many participants mistook the drug for adrenaline and were unclear about its mode of action. The study explored whether such lack of knowledge was related to length of heroin using career as it is possible that knowledge increased with greater experience of the heroin using culture. However a link was not apparent as demonstrated by the account of the following participant who had a 10 year history of heroin use and clearly viewed naloxone as having a role in cardiac rather than respiratory resuscitation:

"If it's heroin and their heart has stopped they will give them adrenaline and just get them breathing again." (Karen, 31 years, 10 year history, council accommodation)

#### Attitudes towards peer use

On establishing users' level of knowledge and awareness of naloxone, participants' attitudes were explored towards the possibility of whether as high risk users they would be willing to carry THN and administer it to a peer in the event of a life-threatening overdose. A clear theme emerged of willingness to administer THN in an emergency situation if required:

"It wouldn't be a problem, I would give it straight away. You have two choices – you have got either the antidote and bring them round and let them live or just sit there and watch them die. It's no choice really." (Steve, 32 years, 12 year history, sleeping rough)

Willingness to administer naloxone appeared to be related to perception of risk taking behaviour by an intimate partner. This participant was keen to have access to THN due to his ongoing fears and worries that his girlfriend would one day die as a result of heroin. In the light of this, naloxone was perceived as an important life saving tool which he could use in the inevitable event of an overdose:

"I would love to have it because if anything like that happened I would love to be able to have the necessary equipment to save her. Because I think one day, the way she uses she will go over one day. She is killing herself now. It hurts me to watch her when she is digging." (Mike, 58 years, 12 month history, hostel accommodation)

Although participants tended to express positive views regarding the potential of peer use of THN, for some this was dependent upon adequate training. Without training there was a perception that either the use of needles or administering a prescribed drug by untrained users would increase the risk to the recipient. Participants feared most the possibility of being open to charges of involuntary manslaughter if giving the drug with good intentions proved fatal despite the best of intentions:

"I'm not going to administer it to somebody who's overdosed, I could end up killing them. I wouldn't personally use it unless I was trained to use it, then you'd know how to use it." (Nick, 29 years, 13 year history, sleeping rough)

"Well if they are not medically qualified and they have got needles, syringes and anything. There is a risk with that." (Sarah, 22 years, 4 year history, private accommodation)

Others perceived a risk to themselves of being in a position of peer administering as reducing their chances of moving away from a heroin using career. They clearly felt that involvement in a drug using culture would be necessary to administer THN effectively and that such involvement was a significant barrier to them achieving a goal of abstinence:

"I would hope not to be carrying it because I don't want to be in that environment with people using and injecting because I am trying to get clean." (Sarah, 22 years, 4 year history, private accommodation)

Negative attitudes of THN within our sample also tended to focus on the potential for precipitating acute heroin withdrawal by the use of THN. Current use of naloxone by clinicians in the emergency setting entails titrating the dose of naloxone against recovery from coma. Administering a single stat dose in the event of a heroin overdose runs the risk of precipitating withdrawal in addition to reversing the heroin induced coma. This participant was clearly able to perceive such a risk of withdrawal by appropriately equating the required dose of THN to be dependent upon the amount of heroin that had been taken prior to the overdose event:

"I dunno. I dunno. Because like surely the amount of, what is it called – Naloxone? The amount of Naloxone that you use is gonna depend on how much heroin the person's used, isn't it? So I dunno." (Alan, 21 years, 4 1/2 year history, probation hostel)

Our findings also demonstrated that participants' willingness to administer THN was situation dependent. Users who would be willing to administer the drug in the event of an accidental overdose reported being less willing to administer the drug if they were aware that the potential recipient had active suicidal intent:

"I would if I knew it were an accident. Like if someone said to me I'm going to have a heroin overdose to top myself then I wouldn't give it to them. But if someone I knew had made a mistake I would." (Mark, 37 years, 20 year history, staying with a friend)

#### Inappropriate use

Naloxone is an opiate antagonist and therefore has no addictive potential as it does not provide the user with a euphoric effect. However concerns have been expressed regarding the theoretical possibility of naloxone being subject to either abuse or malicious use [11,15]. Abuse is the situation in which users take a quantity of heroin in excess of their average intake to maximise the euphoric effect. The theoretical concern is that users' risk perception of the risk of overdose could be reduced if they were aware that a third party would administer naloxone should the excess quantity of heroin precipitate respiratory coma. Malicious use is the situation in which naloxone is forcibly administered to a heroin dependent user who is not in a state of heroin induced coma. Rather the user is either intoxicated, or alert yet not in a state of withdrawal. Administration of naloxone without consent in such a situation precipitates the uncomfortable state of acute withdrawal from heroin.

Participants' attitudes towards the potential for abuse of THN were explored. Although views were expressed that there was a potential for abuse they did not appear to be grounded in users' current experience of heroin using culture:

"They might be tempted to go high because they can give themselves that (Naloxone) but they might not realise or have time to give themselves that." (Neil, 37 years, 2 year history, private accommodation)

Rather the over-riding theme was one of user reluctance to abuse naloxone. The reasons for such reluctance varied. Experience of poverty and the fact that reversing the effects of heroin would be a waste of financial resource were reasons given why abuse of naloxone would be unlikely:

"I couldn't see people just deliberately use loads for the sake of it because it is just a waste of money at the end of the day. If you go over from that jab you've wasted eighty quid haven't you." (Alan, 21 years, 4 1/2 year history, probation hostel)

Also the desire of the heroin user to avoid withdrawal symptoms emerged as a key theme. Abuse of naloxone was identified as putting the user at risk of acute withdrawal. A clear contrast was described between the uncomfortable state of withdrawal and the purpose for taking heroin to alleviate discomfort through the pleasurable euphoric effect:

"People who are addicts take heroin to get rid of pain. It would defeat the purpose of having it in the first place. They wouldn't want to be taking it to do a severe withdrawal afterwards." (Kathryn, 39 years, 26 year history, hostel)

"It wouldn't get abused. I can't see anyone doubling up on their gear because they know they have Narco because the last thing a heroin addict wants is to be injected with something like that or have a blocker dropped on them. You just don't want that to happen." (Peter, 34 years, 21 year history, living with his sister)

Similarly, on exploring the possibility of malicious use of naloxone, some respondents thought that there was a theoretical risk for naloxone to be used maliciously by peers to precipitate an uncomfortable state of withdrawal:

"Because it is the same with everything. You just get your idiots who mess about. I know for a fact there are certain people that would go around and inject people with it just for a laugh just because they could." (Steve, 32 years, 12 year history, sleeping rough)

However despite such certainty, such beliefs were not grounded in previous experience. Rather participants drew parallels with the current situation whereby drug users currently have access to the opiate antagonist naltrexone (referred to by drug users as "blocker"). This medication in tablet form is available to drug users by prescription. However participants consistently reported that the theoretical potential for malicious use of naltrexone was not realised:

"There are blockers on the street, I have a pack of blockers at home and I know of a lot of people who have. So I mean you could go round putting them in each other's tea if you wanted to and that doesn't happen." (Matthew, 34 years, 15 year history, friend's accommodation)

#### Impact on seeking appropriate medical care

The need to call the emergency services following peer administration of THN was explored. As intramuscular naloxone has a short half-life, there is a risk of THN resuscitated drug users slipping back into respiratory coma after the administered naloxone ceases to have a pharmacological effect. Where THN programmes operate in New Mexico, users are still encouraged to attend health services following resuscitation due to the risk of further coma.

A complex picture emerged whereby participants were clearly able to see a role for THN amongst some of the most marginalised of homeless people who tend to be excluded from health services. They described situations in which the risk of heroin overdose is high. This is due to both risk taking in injecting practice and a reluctance to call emergency services. Of concern was the fact that participants tended to idealise the potential of THN. They saw it as obviating the need to call for emergency services, rather than as a safety net to save life whilst emergency attention was sought:

"But I think for people who are like using in a squat or using with a group of people and they might not be too keen on calling an ambulance out or getting help I think that it would be a good idea because there are a lot of people dying from overdoses." (Sarah, 22 years, 4 year history, private accommodation)

Users expressed ambivalence towards the need to call surrounding the need for medical care. Often users expressed awareness that an overdose required medical intervention with some suggesting that Naloxone administration would be sufficient, with no need for follow up care. Referring to the need to attend an accident and emergency department after administration of THN this user reported:

"I think most people would think it's a bit of waste of time. I've had this antidote which is why we would be going to casualty anyway." (Sarah, 22 years, 4 year history, private accommodation)

#### Current information & educational needs

A programme of THN amongst homeless drug users would need to be accompanied by a process of health promotion to enhance knowledge, awareness and personal responsibility. Participants' attitudes towards current health promotion initiatives were explored. They described current health promotion initiatives as being in the form of leaflets and posters. However, a clear narrative emerged that such material was largely ornamental and irrelevant, not directly tailored to their needs:

"No-one reads it. It just looks pretty doesn't it? People don't actually read that stuff. I mean they put it in a shiny wrapper but they're still not going to use it are they? I wouldn't read them, me mum would read them." (Nick, 29 years, 13 year history, sleeping rough)

Also professionally led health promotion initiatives appeared to lack credibility amongst the target population. They were perceived as lacking an understanding and awareness of the use of heroin and offering general advice not specifically tailored to the needs of individuals:

"There are some people who will come and sit and preach to you and they've never seen a bag of smack before, and they are trying to tell you all the symptoms that you have are off a rattle and they don't fucking know, do you know what I mean? They do not know themselves. Fair enough, they might have read a little book detailing what someone else has said but a rattle's different for every person. I dunno, it's just the one thing that I cannot stand – someone who doesn't know shit trying to tell you about it and you're sitting there and you're more clued up than them and there's just no point in it." (Alan, 21 years, 4 1/2 year usage, probation hostel)

Participants revealed that information from fellow users rather than professionals was perceived as more likely to increase their knowledge and awareness. Our research revealed the social interactions of homeless drug users as a situation that could lend itself to peer health promotion activity:

"I have just been told what to do (in the event of an overdose). You just hear what to do; you know what to do kind of thing, we chat and that. You hear what to do or what they say they have done." (Mark, 37 years, 20 year history, living with a friend)

However, despite learning from their peers many users still felt that there was a role for professional input into overdose prevention strategies.

"The majority of people don't know even know what the recovery position is. They have heard of it but they don't know how to do it, so no I don't think it's [information] made available enough." (Kat, 39 years, 26 year habit, hostel accommodation)

## Discussion

We are not aware of any previous qualitative research amongst homeless drug users exploring the concept of THN. Therefore we consider many of the findings of this study to be valuable in understanding how this group can be involved in change. Key findings would suggest that THN could be made more widely available to some homeless drug users. These findings included ability to recognise the signs of an overdose; a willingness to attempt resuscitation (particularly if it involved a partner or significant other); a willingness with training and support to administer THN in some situations, and a belief that abuse or malicious use of THN would not occur as it contravened the social norms of homeless drug using groups. However our findings identified significant barriers which raise question marks as to the effectiveness of an "over the counter" model of provision for homeless drug users. These included a need for greater knowledge and awareness of both THN and wider techniques of resuscitation; a fear that administering THN could precipitate acute withdrawal; a reluctance to give THN where there was known suicidal intent by the intended recipient; fears of killing the recipient with THN; and the failure of current health education literature to effect behaviour change.

### Towards a health promotion model for take home naloxone

Health promotion has been defined as "the process of enabling people to increase control over, and to improve, their health"[22]. However, health promotion has often been used interchangeably with health education and this has reduced its effective application in clinical practice. Our findings suggest that this has been the case with health promotion initiatives aimed at drug users to prevent drug related deaths. Therefore, new frameworks for effective delivery of health promotion initiatives for THN will need to be developed. It is possible that collaborative work with drug users right from the planning stages of such a programme could increase its relevance to and acceptance by homeless drug using populations.

Our findings would suggest that many homeless drug users would have both the motivation and skills to become actively involved in a peer programme of training and distribution of THN. However, in order for this to be achieved, their fears pertaining to involuntary manslaughter need to be addressed. The recent Department of Health Order pertaining to naloxone provides some release from liability providing naloxone is provided within its status as a prescription only medicine. This precludes provision of THN in an "over the counter" model. However it would mean that THN could be provided "on prescription" by both specialists and general practitioners with a special interest in the management of drug use. However such an intervention would only reach drug users actively engaged in treatment. Therefore additional mechanisms would be required to target drug users outside of the treatment setting who are most at risk of drug related death. We would propose that such programmes could be nurse or pharmacist led with THN administered for peer use according to patient group directions (PGDs). PGDs provide the legal and professional framework for prescription only medicines being dispensed by professionals other than doctors [23]. However the direction needs to be developed and signed-off by a senior doctor (specialist or general practitioner with a special interest in drug misuse) and a senior pharmacist. Once developed a PGD would allow nursing or pharmacy staff to train drug users in the techniques of THN. Once trained and deemed competent, nursing or pharmacy staff could then grant a supply of THN on a named patient basis. This model is in fact employed in the USA [8] where a database is kept of users who have been provided with naloxone. It would be important that such governance procedures be kept confidential from police authorities given the fears that homeless people expressed of police involvement when one of their peers has a heroin overdose.

In conclusion previous quantitative cross-sectional studies (where the sampling frame has not been limited specifically to homeless drug users) have shown that despite lacking knowledge about effective CPR resuscitation, drug users still have a sense of responsibility for the well being of their peers [24]. The majority of drug users would be willing to administer naloxone to another person should the need arise [25]. Our findings in concurring with this have further illuminated how such a sense of responsibility can be channelled effectively in services engaging with drug users in the community. We would argue that though provision of THN "over the counter" is practiced in some centres internationally such a model could have limited uptake for homeless people given their expressed need for training regarding appropriate administration of naloxone. Rather it would appear that provision by drug treatment services in an environment confidential from police involvement would have greater potential for uptake. Such provision within a comprehensive package of peer training supported by clear records of when, where and to whom naloxone had been provided is more likely to ensure that the public health benefits outweigh the risks.

## Competing interests

The author(s) declare that they have no competing interests.

## Authors' contributions

NW had the original idea for the study, provided overall project management, was the principal investigator and wrote the first draft.

NO completed the necessary documentation to ensure ethical approval was granted, conducted the interviews and undertook data analysis. Once the first draft had been written she provided input to subsequent drafts.

KF undertook an independent analysis of the data. Once the first draft had been written she provided input to subsequent drafts.

LJ provided supervision of NO as she undertook the process of data analysis and once the first draft had been written she provided input to subsequent drafts.
